# Sociodemographic and health predictors of adherence to self-administered computerized cognitive assessment

**DOI:** 10.1177/20552076251332774

**Published:** 2025-04-10

**Authors:** Marisa Magno, Ana Isabel Martins, Joana Pais, Vítor Tedim Cruz, Anabela G Silva, Nelson Pacheco Rocha

**Affiliations:** 1 Department of Medical Sciences, 56062 University of Aveiro, Aveiro, Portugal; 2 Center for Health Technology and Services Research – CINTESIS@RISE, School of Health Sciences, 56062 University of Aveiro, Aveiro, Portugal; 3 EPIUnit – Institute of Public Health, Laboratory for Integrative and Translational Research in Population Health (ITR), 26706 University of Porto, Porto, Portugal; 4 Neuroinova, Lda., Vila Nova de Gaia, Portugal; 5 379989 Local Health Unit of Matosinhos, EPE, 37824 Hospital Pedro Hispano, Matosinhos, Portugal; 6 IEETA – Institute of Electronics and Informatics Engineering of Aveiro, Department of Medical Sciences, University of Aveiro, Aveiro, Portugal

**Keywords:** Cognitive screening, self-administered computerized cognitive assessment, Brain on Track, general population, adherence, sociodemographic and health predictors

## Abstract

**Introduction:**

Cognitive assessment is essential to detect early cognitive decline and guide interventions. Self-administered computerized assessment is a promising option for periodic cognitive screening in the general population. One of the most critical challenges to implementing cognitive screening in at risk populations is participants’ adherence. However, there is insufficient evidence to determine which factors are essential for adherence to long-term digital cognitive screening.

**Aims:**

This study aims to investigate potential sociodemographic and health predictors of adherence to a self-administered web-based cognitive monitoring, the Brain on Track (BoT), in the general population.

**Methods:**

Participants (*n* = 347) were recruited from the general community. The participants were asked to perform one BoT test every 3 months for cognitive screening and were followed at two time points, namely, 1-year and 3- to 6-year follow-up. Regression models were used to investigate sociodemographic and health predictors of adherence to BoT use at 1 year and up to 6 years.

**Results:**

Being older positively affects adherence to periodic cognitive screening for both follow-up periods. Being a female, having more years of formal education, presenting more BoT baseline correct answers and fewer BoT baseline incorrect answers, and reporting memory complaints positively affect adherence to periodic screening at 3 to 6 years of follow-up but not at 1-year follow-up.

**Discussion:**

The identified determinants of adherence can be considered when planning long-term cognitive screening protocols to increase adherence. Specific strategies could be helpful to improve the adherence of participants who adhere less.

## Introduction

Dementia is a major neurocognitive disorder with significant cognitive decline in one or more cognitive domains and a significant loss of independence in everyday activities.^1^ Due to population aging, the number of people with dementia is expected to increase in the next decades.^2,3^ Worldwide, it was estimated that in 2019, there were more than 50 million people with dementia and this value is expected to rise to 153 million in 2050.^4^

Mild cognitive impairment (MCI) is considered an intermediate stage between normal cognitive aging and dementia and constitutes an increased risk of developing dementia.^5^ It is defined by a modest cognitive decline in one or more cognitive domains (e.g., memory, attention, language, or executive function) without loss of independence in daily activities.^1,6^ The estimated prevalence of MCI ranges from 5.0% to 36.7%^7^ rising with increased age.^8^ However, with an early diagnosis and adequate intervention, it is possible to delay the deterioration of cognitive function in individuals with MCI.^9–11^

Cognitive assessment is essential for identifying cognitive decline at early stages and initiating and guiding intervention.^12–15^ Early detection of MCI has been considered crucial for improving clinical care and assuring proactive, patient-centered management.^8^ An extensive evaluation of multiple cognitive domains performed by a trained professional is the gold standard of cognitive assessment.^5^ However, this approach is not fit for long-term cognitive monitoring of the general population because it is time and resource-consuming^16,17^ and usually involves instruments such as the Mini-Mental State Examination (MMSE)^18^ and the Montreal Cognitive Assessment (MoCA),^19^ which are prone to learning effects on repeated assessments.^20,21^ In the context of cognitive assessment, a 1-year follow-up has been considered clinically meaningful since this period is large enough to enable cognitive monitoring and the detection of cognitive changes over time.^17,22,23^ Therefore, there is the need for novel approaches that can monitor cognitive function over extended periods.^16,24^

Self-administered computerized assessment is emerging as an appealing opportunity for cognitive screening in the general population. It can be conveniently used independently at home,^16,25,26^ periodically repeated since it can incorporate the use of random elements and alternate sequences to minimize learning effects,^16^ and it is cost-effective^16^ and cost-efficient,^27,28^ while allowing the screening of larger numbers of individuals.^26,29^ Nevertheless, the use of self-administered computerized cognitive assessment also has challenges. One of the most important is the low adherence of participants to long-term cognitive monitoring.^25^ For example, Jongstra et al.^29^ found a 60% adherence of participants to a self-administered computerized cognitive assessment over a 6-month period. Therefore, determining which factors could explain low adherence to self-administered computerized cognitive assessment is of uttermost relevance. To our knowledge, no study has specifically investigated the determinants of adherence to self-administered computerized cognitive assessment. Turunen et al.^30^ presented one of the few studies we identified that investigated the determinants of adherence to computerized cognitive training. They examined several cognitive, demographic, lifestyle, and health-related variables as possible predictors and concluded that previous computer use was the only significant determinant of adherence. Another study, Tullo et al.^31^ investigated whether age, gender, cognitive capability, grit, ambition, personality, self-perceived cognitive failures, socioeconomic status, exercise, and education predicted adherence to cognitive training, and reported no association. In contrast, Harrell et al.^32^ reported that measures of memory and self-efficacy were able to predict participants’ adherence to computerized cognitive training. There are other variables that, theoretically, might have a role in predicting adherence to computerized cognitive interventions or assessment, such as lower educational level and increased perceived risk for dementia, including factors associated with dementia such as diabetes, obesity, hypertension, smoking, traumatic brain injury, and depression as risk factors for dementia,^33,34^ dyslipidemia,^35^ anemia,^36^ hypothyroidism,^37^ and hyperthyroidism,^38^ as they can motivate participants to engage in cognitive screening and training continuously. However, we could not find evidence investigating an association between these factors and adherence to cognitive screening.

This study aimed to investigate whether sociodemographic (sex, age, years of education) and health factors (performance in the cognitive screening test, memory complaints, diabetes, obesity, hypertension, current smoking, dyslipidemia, anemia, hypothyroidism, hyperthyroidism, traumatic brain injury, depression) are associated with adherence to self-administered computerized cognitive assessment in the general population at 1-year follow-up and at 3- to 6-year follow-up.

## Methods

This study is a cohort study conducted in Águeda, Portugal, between 2015 and 2021. The study was approved by the Ethics Committee of the Centro Hospitalar de Entre o Douro e Vouga (process number CA-0114/16-0c) on November 25, 2015. To enter the study participants had to provide written informed consent.

### Participants’ recruitment and inclusion criteria

A community-based, non-randomized sample of residents of the region of Águeda, Portugal was used. Participants were recruited between 2015 and 2018 through advertisements in the local media, leaflets placed at key points in the community, and direct invitations at several institutions linked to public administration, industry, education, and social services. To be included in the study, participants had to meet the following inclusion criteria: (i) be 18 years old or older; (ii) be able to use the mouse/computer interface autonomously; (iii) have access to a computer and Internet; and (iv) have at least one complete year of schooling. Participants were excluded if reporting: (i) severe motor, visual, or language deficits that prevented cognitive assessment and (ii) were unable to read. All participants who completed the assessment at the baseline, including sociodemographic and health factors and cognitive functioning, entered the study. Follow-up occurred until 2021. Thus, participants were followed for a minimum of 3 and a maximum of 6 years.

### Participants’ baseline assessment

The baseline assessment was conducted in person. Participants who entered the study responded to a sociodemographic and health-related questionnaire. Data was collected using a questionnaire developed by the current study researchers that collected self-reported information on sex, age, years of education, memory complaints and use of medication to improve memory, presence of risk factors for cardiovascular disease (e.g., diabetes, obesity, hypertension, current smoking), and presence of other comorbidities (e.g., dyslipidemia, anemia, hypothyroidism, hyperthyroidism, traumatic brain injury, depression). Each of these variables was assessed with a yes/no question. The questionnaire was specifically developed for the present study and was not subject to previous validation (Supplementary file).

Cognitive functioning was assessed using the digital solution Brain on Track (BoT). This is a self-administered web-based cognitive digital solution that includes a pool of different exercises within each of eleven subtests (e.g., word categories, sequences, puzzles, inhibitory control tasks, verbal memory tasks, delayed verbal memory tasks, and attention tasks) that evaluate six cognitive domains (memory, attention, language, calculus, executive functions, and constructive ability).^16,17^ Developed for screening and repetitive use, the BoT randomly generates different questions and alternate sequences at each assessment to minimize learning effects. Also, the subtests of BoT include various versions with different difficulty levels, allowing the adjustment of subtests to the performance of participants based on academic level.^16^

The duration of the subtests and the response time define the number of tasks presented to the participants in each assessment subtest. The score of each subtest equals the number of tasks correctly performed^16^ and the total score can vary between 0 and the maximum number of tasks that participants can complete correctly within the time limit. The number of tasks presented to each person depends on the participant's performance within the fixed time limit. Therefore, the maximum score is not fixed and varies from person to person.

The number of tasks incorrectly performed was also collected since participants have immediate feedback if one task is correctly or incorrectly answered, which can affect test adherence. Confidence in the ability to succeed has been identified as a critical factor in adherence to non-pharmacological interventions.^39^ Thus, for this study, the following variables related to the performance of the BoT test were used: total score of BoT at baseline (i.e., the sum of the number of tasks correctly realized in each subtest, with higher scores indicating better cognitive performance); total of incorrect answers given in BoT at baseline (i.e., the sum of the number of tasks incorrectly realized in each subtest, whose result is not counted for the score of BoT test). Both BoT at baseline correct answers and BoT at baseline incorrect answers were included as predictors in the regression models, as they assess different aspects of performance. While the first captures the total number of correct responses, the second is related to the number of incorrect responses given by participants. Since there is no fixed total number of responses, one variable does not directly determine the other.

Participants were asked to perform one BoT test every 3 months for cognitive screening for a minimum of 3 and a maximum of 6 years. After the first assessment, participants received no further encouragement to use the BoT. The number and date of BoT tests performed by each participant was also collected.

### Time points and indicators of adherence

Two time points were defined for the study of adherence to self-administrated computerized cognitive assessment: (i) 1-year follow-up (the first year after enrolling in the study), considering that this period is clinically meaningful, being large enough to enable cognitive monitoring and the detection of cognitive changes^17,22,23^; (ii) 3- to 6-year follow-up (i.e., the period of follow-up of each participant varied between 3 and 6 years), considering the need and relevance of monitoring cognitive function over extended periods.

At 1-year follow-up, adherence was defined as having used the BoT at least four times (out of the five recommended times) during the first year of study enrollment (from baseline). Thus, participants’ adherence at the 1-year follow-up was defined as a binary variable: non-adherent (less than four BoT tests performed within the first year from baseline) and adherent (at least four BoT tests performed within the first year from baseline). This threshold was chosen because it aligns with a clinically meaningful period for cognitive monitoring. It distinguishes between participants who adhered to the recommended assessment and those who did not.

At 3- to 6-year follow-up no cut-off was used to define adherence as the number of BoT tests performed by each participant was used as the dependent variable in the analysis.

### Statistical analysis

Mean and standard deviation and count and proportion were used to describe quantitative and qualitative variables, respectively. The study of adherence to BoT was investigated for the two time points previously described.

A logistic regression model was used to investigate the sociodemographic and health predictors of adherence at 1-year follow-up, considering adherence to BoT if participants had performed the BoT at least four times during 1 year (from baseline). The dependent variable studied was binary (0 = no adherence to the BoT test, i.e., less than four times of use during 1 year from baseline; 1 = adherence to the BoT test, i.e., at least four times of use during 1 year from baseline). Its association with the following independent variables was explored in univariable logistic regression analysis: sex (0 = female, 1 = male); age; years of education; total of BoT correct answers at baseline; total of BoT incorrect answers at baseline; memory complaints and/or use of medication to help memory (0 = no complaints, 1 = memory complaints and/or medication use); risk factors for cardiovascular disease—diabetes, obesity, hypertension, and/or current smoking (0 = no risk factors reported, 1 = 1 or more risk factors reported); other comorbidities—presence of dyslipidemia, anemia, hypothyroidism, hyperthyroidism, traumatic brain injury and/or depression (0 = no comorbidities, 1 = 1 or more comorbidities). Thus, the information regarding health factors was scored zero (in case of absence) or one (in case of presence).

Independent variables with a *p *≤ 0.10 on univariable logistic regression analysis were included as predictors in the multivariable logistic regression model to investigate BoT adherence. The link function used in the logistic regression model was the logit function.

For the investigation of adherence to BoT, using the number of BoT tests performed during 3- to 6-year follow-up as the dependent variable, a Poisson regression model was used to investigate the sociodemographic and health predictors.

Independent variables with a *p* ≤ 0.10 on univariable Poisson regression analysis were included as predictors in the multivariable Poisson regression model. Time using the BoT was entered as an offset variable as not all participants were using the BoT for the same amount of time. Thus, there was the need to adjust the dependent variable and independent variables to different follow-up durations. Poisson distributed data is inherently integer-valued, which is the case for this count data (dependent variable can take integer values ≥ 1), and to scale the modeling to time, Poisson regression allows using an offset variable.

The multivariable analyses were performed using the enter model, and the independent variables were checked for multicollinearity using variance inflation factor (VIF) ≤ 5. The level of significance was set at *p* < 0.05.

In the linear regression models, the calculation of *R^2^* and *R^2^* adjusted indicates the proportion of variance explained by the models. In logistic and Poisson regression models, the interpretation differs. In logistic regression, the proportion of explained variance is given by pseudo *R^2^*, with measures such as Nagelkerke's *R^2^*, which is an adjusted version of *R^2^* calculated for the multivariable model and for the univariable models whose variables entered in the multivariable model.^40^ For Poisson regression, *R^2^* was also calculated. However, its interpretation differs, as it assesses the adjustment quality using the residuals’ deviations.

Post hoc sample size calculations were conducted to assess whether the sample size was adequate to detect the effects of the predictors included in the multivariable regression models at the 1-year and 3- to 6-year follow-up assessments. The calculations were performed with a significance level of 0.05, a power of 0.80, and a small to medium effect size.

Statistical analyses were performed using R (R Core Team, Vienna).

## Results

### Sample characteristics and number of tests taken

This study included 347 participants, 249 (71.8%) of whom were females. The mean age was 46.9 (SD 12.7) years old (range 25–81 years). The characteristics of the study participants when entering the study are presented in Table 1.

**Table 1.** Characteristics of participants at baseline (*n* = 347).

Variables	
Sex, *n* (%)	
Female	249 (71.8%)
Male	98 (28.2%)
Age, mean (± SD)	46.9 (±12.7)
Years of formal education, mean (± SD)	14.1 (±3.8)
Brain on Track (BoT) at baseline (correct answers), mean (± SD)	211.3 (±60.6)
Brain on Track (BoT) at baseline (incorrect answers), mean (± SD)	10.6 (±12.4)
Reporting memory complaints, *n* (%)	85 (24.5%)
Reporting of at least one cardiovascular risk factor, *n* (%)	100 (28.8%)
Diabetes, *n* (%)	10 (2.9%)
Obesity, *n* (%)	22 (6.3%)
Hypertension, *n* (%)	44 (12.7%)
Current smoking, *n* (%)	44 (12.7%)
Reporting at least one comorbidity, *n* (%)	138 (39.8%)
Dyslipidemia, *n* (%)	35 (10.1%)
Anemia, *n* (%)	33 (9.5%)
Hypothyroidism, *n* (%)	15 (4.3%)
Hyperthyroidism, *n* (%)	13 (3.7%)
Traumatic brain injury, *n* (%)	5 (1.4%)
Depression, *n* (%)	83 (23.9%)

**Figure 1.**
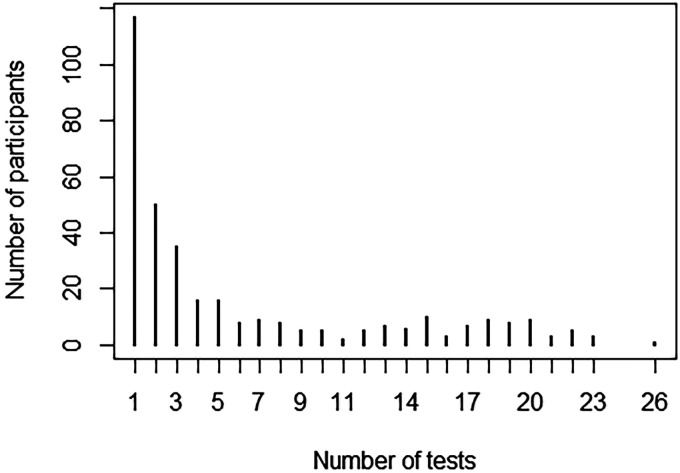
Distribution of the number of BoT tests performed by participants during the 3- to 6-year follow-up.

The mean (±SD) number of BoT tests performed by each participant during the entire follow-up period (3–6 years) was 6.0 (SD 6.6), ranging from 1 to 26 tests. One hundred and seventeen participants (33.7%) completed only one BoT test (at baseline), 101 (29.1%) completed 2 to 4 tests, 41 (11.8%) completed 5 to 8 tests, 17 (4.9%) completed 9 to 12 tests, 26 (7.5%) participants completed 13 to 16 tests, and 45 (13.0%) completed more than 17 tests. The distribution of the number of tests performed by participants is presented in Figure 1.

### Predictors of 1-year adherence to cognitive screening

Regarding the 1-year adherence to self-administrated computerized cognitive assessment, 125 participants (36%) used the BoT at least four times during the first year of study enrollment, and 222 participants (64%) did not (i.e., used the BoT less than four times).

In the univariable logistic regression models for predicting participants’ adherence to 1-year BoT use, age, memory complaints, and other identified comorbidities, were significant at *p *< 0.1 and were included as predictors in the multivariable logistic regression model to investigate BoT adherence (Table 2).

**Table 2.** Univariable and multivariable logistic regression models for predicting adherence to Brain on Track at 1-year follow-up (at least four times of use during 1 year from baseline).

	Univariable				Multivariable			
Variables	Coefficient *B* (95% CI)	Odds Ratio (95% CI)	*p*	*R* ^2^	Coefficient *B* (95% CI)	Odds Ratio (95% CI)	*p*	*R* ^2^
Sex (male)	−0.335 (−0.845; 0.158)	0.715 (0.430; 1.171)	0.189					0.08
Age	0.037 (0.019; 0.056)	1.038 (1.020; 1.057)	<0.001*	0.07	0.033 (0.014; 0.052)	1.033 (1.014; 1.053)	<0.001*	
Years of formal education	0.022 (−0.036; 0.081)	1.022 (0.965; 1.084)	0.459					
Brain on Track (BoT) first test correct answers	−0.001 (−0.005; 0.003)	0.999 (0.995; 1.003)	0.604					
Brain on Track (BoT) first test incorrect answers	−0.010 (−0.034; −0.008)	0.990 (0.966; 1.009)	0.332					
Memory issues (yes)	0.486 (−0.015; 0.985)	1.626 (0.985; 2.678)	0.056	0.01	0.257 (−0.279; 0.785)	1.293 (0.757; 2.193)	0.343	
Cardiovascular risk factors (yes)	−0.001 (−0. 491; 0.479)	0.999 (0.612; 1.614)	0.995					
Others identified comorbidities (yes)	0.637 (0.191; 1.086)	1.891 (1.210; 2.962)	0.005*	0.03	0.386 (−0.096; 0.866)	1.471 (0.908; 2.377)	0.116	

* Statistically significant, *p*-value < 0.05.

Only the independent variable age was significant in the multivariable logistic regression model for predicting adherence to BoT (Table 2). Results showed that being older positively affects the participants’ adherence to BoT. The multivariable model revealed an *R^2^* of 0.08 (Nagelkerke R-squared measure), which is similar to the *R^2^* of 0.07 (Nagelkerke *R^2^*) from the univariable model with age, indicating a limited explanatory capacity.

### Predictors of 3 to 6 years adherence to cognitive screening

In the univariable Poisson regression models for predicting participants’ adherence to BoT, including the 347 participants, all independent variables, except cardiovascular risk factors, were statistically significant at *p *< 0.05 (Table 3) and were included in the multivariable Poisson regression model.

**Table 3.** Univariable and multivariable Poisson regression models for predicting adherence to Brain on Track at 3- to 6-year follow-up (number of tests realized).

	Univariable				Multivariable			
Variables	Coefficient *B* (95% CI)	Risk Ratio (95% CI)	*p*	*R^2^*	Coefficient *B* (95% CI)	Risk Ratio (95% CI)	*p*	*R^2^*
Sex (male)	−0.208 (−0.310; −0.107)	0.812 (0.733; 0.899)	<0.001*	0.008	−0.236 (−0.350; −0.124)	0.790 (0.705; 0.883)	<0.001*	0.17
Age	0.022 (0.019; 0.025)	1.022 (1.019; 1.025)	<0.001*	0.08	0.036 (0.032; 0.041)	1.037 (1.033; 1.042)	<0.001*	
Years of formal education	0.013 (0.002; 0.025)	1.013 (1.002; 1.025)	0.0228*	0.002	0.019 (0.006; 0.031)	1.019 (1.006; 1.031)	0.003*	
Brain on Track (BoT) first test correct answers	−0.001 (−0.001; −0.0003)	0.999 (0.999; 0.9997)	0.0059*	0.004	0.004 (0.003; 0.005)	1.004 (1.003; 1.005)	<0.001*	
Brain on Track (BoT) first test incorrect answers	−0.010 (−0.014; −0.005)	0.990 (0.986; 0.995)	<0.001*	0.009	−0.015 (−0.021; −0.009)	0.986 (0.979; 0.991)	<0.001*	
Memory issues (yes)	0.365 (0.272; 0.456)	1.441 (1.313; 1.578)	<0.001*	0.03	0.335 (0.238; 0.433)	1.399 (1.269; 1.542)	<0.001*	
Cardiovascular risk factors (yes)	−0.041 (−0.137; 0.055)	0.960 (0.872; 1.057)	0.408					
Others identified comorbidities (yes)	0.249 (0.162; 0.335)	1.283 (1.176; 1.398)	<0.001*	0.02	0.040 (−0.056; 0.136)	1.041 (0.946; 1.146)	0.4153	

* Statistically significant, *p*-value < 0.05.

The independent variables sex, age, years of formal education, BoT first test correct answers, BoT first test incorrect answers, and memory issues remained significant in the multivariable model for predicting adherence to BoT (Table 3). Results showed that being a female, being older, having more years of formal education, presenting more BoT baseline correct answers and fewer BoT baseline incorrect answers, and reporting memory complaints positively affect the number of BoT tests performed. In the multivariable model, no multicollinearity was detected among the independent variables, supporting their inclusion as distinct predictors. The multivariable model revealed an *R^2^* of 0.17, indicating a better model fit compared to the univariable models.

Post hoc power analysis indicated that the sample size for the multivariable regression models (347 participants) was sufficient to detect meaningful effects of the predictors at the 1-year and 3- to 6-year follow-up, with a power of 0.80 and a small to medium effect size.

## Discussion

The results of the present study showed that being older positively affects adherence to periodic cognitive screening for the period of 1-year. Also, being older, being a female, having more years of formal education, presenting more BoT baseline correct answers and fewer BoT baseline incorrect answers, and reporting memory complaints positively affect the number of BoT tests performed at 3- to 6-year follow-up but not the 1-year adherence to periodic screening.

Our results cannot be directly compared to previous studies as we were unable to find studies exploring factors associated with adherence to self-administered computerized cognitive assessment. Studies exploring adherence to digital interventions for shorter follow-up periods (i.e., maximum of 6-month follow-up) are also scarce and showed conflicting results. Harrell et al.^32^ investigated the factors associated with adherence to a 4.5-month technology-based cognitive intervention and reported that higher memory scores were associated with higher participants’ adherence to technology-based cognitive interventions. Also, Harrell et al.,^32^ Turunen et al.,^30^ and Tullo et al.^31^ did not find an association between adherence to cognitive training using digital solutions and sex and years of formal education for shorter follow-up periods (maximum of 6 months). The contrasting findings of these studies with the current study findings might be due to the different nature of what is being assessed (adherence to screening vs. adherence to an intervention).

Aging is associated with the development of dementia by the general population,^41,42^ and the participant's perceived risk for dementia related to age may contribute to explaining why being older positively affected adherence to cognitive screening in the current study. On the other hand, we did not find any association between the presence of cardiovascular risk factors or other identified comorbidities and BoT adherence at 1 year and at 3- to 6-year follow-up. One possible explanation for these health-related variables not being predictors of adherence to cognitive screening might be that the general population is unaware of the role that cardiovascular risk factors and other comorbidities might play in the development of dementia.^41^ This needs to be further investigated and potentially addressed in campaigns related to health literacy.

Since cognitive screening is necessary for identifying cognitive decline and enabling intervention at an early stage,^12–15^ it seems particularly important that the population at risk for cognitive impairment can benefit from continuous screening of cognitive performance in a long-term follow-up. Several studies have addressed risk factors that contribute to cognitive impairment, and the multiple risk factors are known (e.g., diabetes, hypertension, hypercholesterolemia, depression, physical frailty, low education level, or low social support level), with age being the most important one.^43–48^ Despite our work having identified as possible determinants of adherence aging (at 1-year and 3- to 6-year follow-up) and memory complaints (only at 3- to 6-year follow-up), health-related risk factors for dementia were not associated with higher adherence to cognitive long-term screening. Therefore, adherence to cognitive screening may not be associated with dementia risk factors. As referred previously, the lack of knowledge regarding dementia health-related risk factors in the general population^41^ may explain these findings since there is no perceived risk for participants having cardiovascular risk factors or other comorbidities that could enhance adherence to BoT. On the other hand, memory loss is recognized by the general population as being related to dementia,^42^ which may contribute to explaining the positive association that we found between the presence of memory issues and the number of BoT tests performed by participants in the long-term period, namely, at 3- to 6-year follow-up. Participants who presented memory issues may be aware of their high risk for dementia, which may enhance their adherence to BoT in a long-term follow-up period.

Concerning sex differences, the prevalence of dementia differs with sex, being more prevalent in women than men,^4^ and previous studies pointed out that women are better informed about dementia than men.^42^ Our results revealed that being a female positively affects the number of BoT tests performed at 3 to 6 years of follow-up, which can be related to eventual women's major perceived risk and dementia literacy.

Regarding formal education, our results suggest that participants with more years of formal education are more likely to adhere to long-term online cognitive screening, namely, to a 3- to 6-year follow-up. One possible explanation is that a higher education level is associated with higher health literacy, enhancing the awareness of healthy behaviors and preventive care.^49^ Also, higher education can be related to higher digital literacy, facilitating the use of technology. Turunen et al.^30^ found that previous computer use was the only significant determinant of adherence to a 4- to 6-month computerized cognitive training when examining several cognitive, demographic, lifestyle, and health-related variables as possible predictors. Furthermore, the participants’ confidence in the ability to succeed using technology was found to also contribute to participants’ adherence to digital health solutions.^50^ Our results showed that presenting more BoT baseline correct answers and fewer BoT baseline incorrect answers positively affects the number of BoT tests performed at 3 to 6 years of follow-up. These results can be related to the participant's confidence in the ability to succeed while using BoT since participants have immediate feedback on whether one task is correctly or incorrectly answered, which consequently may affect participants’ adherence.

Discrepancies between the factors associated with 1-year adherence and the number of tests performed during 3 to 6 years follow-up might be due to differences in the definition used (adherence vs. number of tests performed). For the 1-year follow-up, adherence was defined as a binary variable (adherent vs. non-adherent), and a logistic regression model was conducted to identify sociodemographic and health-related predictors of adherence. This approach, distinguished between participants who met the predefined adherence threshold and those who did not, but did not capture variations in the number of tests completed beyond the adherence cutoff. In contrast, for the 3- to 6-year follow-up, adherence was investigated as a count variable, using a Poisson regression model to examine the total number of tests performed over an extended follow-up period. This model is likely to offer a more refined estimation of adherence over time since it modeled a count variable, identifying factors related to test completion frequency and not only a binary adherence status. Therefore, the 3- to 6-year follow-up model identified a broader range of determinants of adherence. These methodological differences and the distinct follow-up periods may contribute to the observed discrepancies in determinants in the two time points of indicators of adherence.

Digital solutions may facilitate the integration of cognitive screening into population-based screening programs, particularly those aiming at the early detection of cognitive impairment.^51^ Self-administered digital tools improve availability and accessibility of cognitive screening, particularly to those individuals with limited mobility or restricted access to specialized healthcare services. Additionally, digital cognitive screening tools offer a cost-efficient solution that can be scaled for widespread use.^27,28^ Investigating the determinants of adherence to self-administered digital solutions is crucial for planning long-term cognitive screening protocols.

### Limitations, research and clinical implications

The regression model showed a limited capacity to investigate the sociodemographic and health predictors that could explain adherence at 1-year follow-up, defined as the presence of at least four times of use during 1 year from baseline. Thus, other variables can affect adherence to self-administrated cognitive screening, such as digital literacy,^30^ which could explain the model's low proportion of explained variance. In future studies, variables related to participants’ digital literacy and access to personal computers and the Internet can be considered for both time points. Also, the role of social and cultural determinants, such as social support, income, social status, healthcare access, and economic, political, and religious systems can also be explored when investigating participants’ adherence to online cognitive screening. Including these additional variables in future studies might enhance the proportion of explained variance.

The results of this study are for a specific digital solution and a community-based context and might not apply to other digital solutions or contexts. Also, due to the non-randomized sampling, potential sampling bias should be considered, as study designs that do not use representative samples of the population, particularly those with low response rates, are at risk of bias.^52^

The questionnaire used in this study to collect self-reported sociodemographic and health-related data was developed for this study and was not subject to previous validation. Self-reported questionnaires are commonly used in research but may be subject to biases, namely, recall bias.^52^ In future research, studies should use validated instruments and objective measures alongside self-reported data.

## Conclusion

The determinants of adherence identified in the current study can be considered when planning long-term cognitive screening protocols for enhancing adherence. Specific strategies could be designed to increase the adherence of participants who potentially adhere less.

## Supplemental Material

sj-pdf-1-dhj-10.1177_20552076251332774 - Supplemental material for Sociodemographic and health predictors of adherence to self-administered computerized cognitive assessment Supplemental material, sj-pdf-1-dhj-10.1177_20552076251332774 for Sociodemographic and health predictors of adherence to self-administered computerized cognitive assessment by Marisa Magno, Ana Isabel Martins, Joana Pais, Vítor Tedim Cruz and Anabela G Silva, Nelson Pacheco Rocha in DIGITAL HEALTH
