# Supplementary material for: Sociodemographic and health predictors of adherence to self-administered computerized cognitive assessment
Source: Digit Health. 2025 Apr 10;11:20552076251332774. doi: 10.1177/20552076251332774 (PMC12032462; doi:10.1177/20552076251332774)
Supplement: sj-pdf-1-dhj-10.1177_20552076251332774 - Supplemental material for Sociodemographic and health predictors of adherence to self-administered computerized cognitive assessment [file sj-pdf-1-dhj-10.1177_20552076251332774.pdf]

ID: ________________

SOCIODEMOGRAPHIC AND HEALTH QUESTIONNAIRE

Sociodemographic Data

Sex: ______________

Age: ______________

Years of formal education (completed): _____________

Health Information

- Have you ever had **memory complaints and/or used medication to improve memory**?

  ☐ **Yes** ☐ **No**

- Have you ever been diagnosed with any of the following conditions?

**Condition**	**Yes**	**No**
**Diabetes**	☐	☐
**Obesity**	☐	☐
**Hypertension**	☐	☐
**Dyslipidemia**	☐	☐
**Anemia**	☐	☐
**Hypothyroidism**	☐	☐
**Hyperthyroidism**	☐	☐
**Traumatic brain injury**	☐	☐
**Depression**	☐	☐

- Do you **currently smoke**?

  ☐ **Yes** ☐ **No**
